# Innovative electronic publication in plant systematics: PhytoKeys and the changes to the “Botanical Code” accepted at the XVIII International Botanical Congress in Melbourne

**DOI:** 10.3897/phytokeys.6.2063

**Published:** 2011-09-14

**Authors:** W. John Kress, Lyubomir Penev

**Affiliations:** 1 Department of Botany, National Museum of Natural History, Washington, U.S.A.; 2 Pensoft Publishers, Sofia, Bulgaria

PhytoKeys was established less than a year ago in response to four main publication challenges of our time: (1) the appearance of electronic publications as amendments or even alternatives to paper publications; (2) Open Access (OA) as a new publishing model; (3) the linkage of electronic registers, indices, and aggregators, which summarize information on biological species through taxonomic names or their persistent identifiers; and (4) Web 2.0 technologies, which permit the semantic markup of, and semantic enhancements to, published biological texts. The appearance of the journal was concomitant with lively discussions on the validity of nomenclatural acts published electronically (Knapp and Wright 2010, Knapp et al. 2010, Penev et al. 2010, Chapman et al. 2010). At the XVIII International Botanical Congress in Melbourne in July 2011 (IBC 2011) these discussions culminated in the decision to amend the *International Code of Botanical Nomenclature* to allow electronic-only publishing of new taxa. Even before the end of the Congress and formal acceptance of the changes PhytoKeys was able to publish a report on the main outcomes of the Nomenclature Section on electronic publishing (Miller et al. 2011).

During the year preceding the IBC 2011, PhytoKeys invested significant effort and resources in preparing the journal’s infrastructure to meet the new challenges of electronic-only publication. PhytoKeys was the first journal to mandate the inclusion of International Plant Name Index (IPNI) identifiers in all original descriptions of new species (protologues) and hence a workflow for *pre-publication registration of nomenclatural acts*. Although the Nomenclature Section in Melbourne declined a proposal for mandatory pre-publication registration for acts in plants and algae, it approved the mandatory registration of fungal names on and after 1 January 2013 (see McNeill and Turland 2011, Hawksworth 2011 and Norvell in press for details). Following its proclaimed policy to always be at the forefront of biodiversity publishing, Pensoft launched MycoKeys, a sister journal to PhytoKeys, which requires mandatory inclusion of the MycoBank registration numbers in the protologues of new species (Lumbsch et al. 2011, Hawksworth 2011).

PhytoKeys has also been at the vanguard of “atomized” content, i.e., to separately distribute the taxonomic information included in a paper to relevant on-line aggregators. Thanks to its advanced XML-based editorial workflow, the journal exports taxon treatments to the Encyclopedia of Life, the Plazi Treatment Repository, and Wiki (Species-ID) on the day of publication. PhytoKeys also provides an established infrastructure for data publishing in cooperation with the Global Biodiversity Information Facility (GBIF) and the Dryad Data Repository.

Another important aspect of electronic publishing of nomenclatural acts is the long-term archival preservation of e-publications. Unfortunately, the Nomenclature Section in Melbourne addressed this question only in Recommendation 29A (see Knapp et al. 2011 in the present volume). Nonetheless, PhytoKeys now has a solution in place for this problem through a successful application for archiving in PubMedCentral, perhaps the most important archive for biomedical literature in the world. Thanks to adoption of TaxPub (www.sourceforge.net/projects/taxpub), an extension of the Journal Archiving and Interchange Tag Suite (JATS) maintained by the U.S. National Center for Biotechnology Information (NCBI) of the U.S. National Library of Medicine (NLM), all papers published in PhytoKeys will be archived in PubMedCentral in three versions, as PDF (the version which constitutes effective publication under the new rules, see Knapp et al. 2011, this volume), HTML, and XML. In addition, all images associated with a paper are stored and indexed in duplicate, as separate files. An additional guarantee for the long-term preservation of publications containing the names of new taxa is the wide dissemination of the open access articles, including the separate deposition of taxon treatments in various aggregators, as mentioned above.

The current issue of PhytoKeys further consolidates the strong commitment of the journal to revolutionizing the landscape of taxonomic publishing. The paper by Knapp et al. (2011) lists those amendments to the *Melbourne Code* that address electronic publication and is being or will be co-published in sixteen different journals (see the publishing statement in Knapp et al. 2011). To expedite the widest possible dissemination of this important paper, PhytoKeys and MycoKeys undertook the translation of the paper into Chinese, Portuguese, Russian, and Spanish and published the translated versions in today’s issues of both journals.

We are extremely pleased to see that most of the policies and publishing practices outlined in the opening paper of PhytoKeys (Penev et al. 2010) have been adopted by the botanical and mycological communities whose deliberations in Melbourne will result in the (newly named*) International Code of Nomenclature for algae, fungi, and plants* (see McNeill and Turland 2011; McNeill et al. 2011). The journal is fully prepared to continue to adapt to the new era of taxonomic publishing. Editorial policies of individual journals will eventually determine how these new changes will affect publishing practice.

In practical terms, PhytoKeys will now adhere to the following editorial policies:

•	Mandate the inclusion of the IPNI registration numbers in the original descriptions (protologues). Authors are not requested to provide registration numbers, as the whole process of registration is provided by the Editorial Office of the journal in collaboration with IPNI
•	Publish each paper in four versions: (1) PDF for effective publication, reference and easy archiving; (2) full-colour, high-resolution print version identical to the effectively published PDF version; (3) HTML for easy reading, browsing and applying semantic enhancements to the text; and (4) XML to provide a machine-readable file for archiving and data mining
•	Produce a print version, identical to the PDF, which will be deposited it in six important botanical libraries of the world: Smithsonian Institution, Washington D.C.; Natural History Museum, London; Royal Botanic Gardens, Kew; Missouri Botanical Garden, St. Louis; Komarov Botanical Institute, St. Petersburg; Kunming Institute of Botany Heilongtan, Kunming, China.
•	Shorten the publication time to a maximum of one to two weeks after the editorial acceptance of a manuscript
•	Continuously develop and implement cutting-edge publishing technologies: XML-based editorial work flow and mark up process, data publication and various semantic Web 2.0 enhancements.

Finally, we would like to thank all of the authors, editors and readers of PhytoKeys for their support of the journal, as well as the translators of the paper of Knapp et al. (2011): Li-Bing Zhang (Chinese), Jefferson Prado, Regina Y. Hirai, and Cíntia Kameyama (Portuguese),Irina Belyaeva and Maria Vorontsova (Russian), and Carmen Ulloa Ulloa, Lourdes Rico Arce, and Renée H. Fortunato (Spanish). Special thanks are due to all teams that made possible the establishment of the innovative workflow of Pensoft’s journals: Plazi, the Global Biodiversity Information Facility (GBIF), the Encyclopedia of Life (EOL), the Biodiversity Heritage Library (BHL), the National Library of Medicine of the U.S. (NLM), and the ViBRANT EU FP7 project. We also thank the staff of IPNI for helping us to establish a workflow for the provision of IPNI identifiers for new species of flowering plants.
